# Active surveillance for low-risk papillary thyroid microcarcinoma: a web-survey on clinician readiness for change

**DOI:** 10.1530/ETJ-25-0013

**Published:** 2025-03-17

**Authors:** Grigoris Effraimidis, Eleni Sazakli, Olga Karapanou, Katerina Saltiki, Marina Michalaki

**Affiliations:** ^1^ Department of Endocrinology and Metabolic Diseases, Larissa University Hospital, Faculty of Medicine, School of Health Sciences, University of Thessaly, Larissa, Greece; ^2^ Faculty of Medicine, School of Health Science, University of Patras, Patras, Greece; ^3^ Endocrine Department, NIMTS Veteran’s Hospital, Athens, Greece; ^4^ Endocrine Unit, Department of Clinical Therapeutics, National and Kapodistrian University, Athens, Greece; ^5^ Endocrine Division, Faculty of Medicine, School of Health Science, University of Patras, Patras, Greece

**Keywords:** papillary thyroid microcarcinoma, active surveillance, web-survey, lobectomy

## Abstract

**Introduction:**

Current guidelines emphasize active surveillance (AS) over immediate surgery for low-risk papillary thyroid microcarcinomas (PTMCs). Alternative minimally invasive treatments, such as thermal ablation, are being explored. If thyroidectomy is performed, lobectomy is preferred and radioactive iodine (RAI) remnant ablation is not routinely recommended for low-risk PTMC patients.

**Aim:**

This study aimed to assess the approach of Greek endocrinologists toward AS and the management of low-risk PTMCs.

**Methods:**

A web-based survey was conducted among members of the Hellenic Endocrine Society (HES). Two clinical scenarios involving a 60-year-old woman with low-risk PTMC were analyzed. Surveyed endocrinologists were asked whether they would recommend AS, thermal ablation, lobectomy or total thyroidectomy as primary treatment; and if total thyroidectomy was performed in this case, whether they would recommend RAI ablation.

**Results:**

A total of 201 endocrinologists (25% of HES members) participated. As primary treatment for low-risk PTMC, 46.8% recommended total thyroidectomy, 31.3% chose AS, 20.9% opted for lobectomy and 1.0% selected thermal ablation. If total thyroidectomy was performed, 95% considered RAI ablation unlikely and only 5% would use RAI. Demographic characteristics, including age, sex, experience and geographic location, did not significantly influence these choices. The primary reason cited by endocrinologists for noncompliance is skepticism about implementing the guidelines, likely stemming from resource limitations and educational gaps.

**Conclusion:**

One-third of Greek endocrinologists prefer AS for managing low-risk PTMCs. More time and effort may be needed to further shift their clinical approach. Insights from our web survey aim to reduce overtreatment in low-risk PTMC management.

## Introduction

Over the past decades, the global incidence of thyroid cancer has risen significantly (1). As of 2022, thyroid cancer ranks as the seventh most common cancer worldwide, with women experiencing a three-fold higher incidence rate compared to men (2). Despite this increase in incidence, the mortality rate remains relatively low and stable over time (3). A study across 25 countries revealed that this surge in incidence primarily involves small, occult papillary carcinomas, often detected through intensified inspection of the thyroid gland due to the widespread use of imaging techniques, including thyroid ultrasonography, and diagnostic tools such as fine-needle sampling (4, 5). These lesions are very unlikely to cause significant morbidity or mortality, falling in the definition of overdiagnosis (6). The overdiagnosis of thyroid cancer has led to unnecessary treatments, imposing significant psychological, behavioral and financial burden on individuals and on society as a whole.

In response to these challenges, recent updates to national and international clinical guidelines emphasize active surveillance (AS) rather than immediate intervention for cases involving papillary thyroid microcarcinomas (PTMCs) (7, 8, 9, 10), defined as tumors having 10 mm or less as maximum diameter. In most cases, PTMC is an incidental finding, identified from specimens removed for benign thyroid conditions (11, 12). Less frequently, it is diagnosed as a suspected subcentimetric thyroid nodule detected during routine neck ultrasonography or CT scans (5, 13).

AS as a management strategy for selected PTMC patients was first introduced in Japan during the 1990s (14, 15). It consists of closely and regularly monitoring the disease status of the patient and offering rescue surgery in the case disease progress is detected. Since the first studies, other clinical trials have demonstrated its feasibility, leading to the global adoption of AS in countries including Japan, South Korea, the United States, Italy, Argentina and Brazil (16, 17, 18, 19). In 2015, the American Thyroid Association (ATA) formally recognized AS as a management alternative to lobectomy for patients with low-risk PTMC (7). Despite its growing acceptance, concerns remain about the long-term AS, particularly in younger patients, anxious and worried patients, patients lost to follow-up and questions about its cost-effectiveness. In light of these challenges, ultrasound-guided percutaneous ablation techniques, such as thermal ablation, have emerged as promising alternatives to surgery for managing very low-risk and low-risk PTMC (20).

Radioactive iodine (RAI) remnant ablation following total thyroidectomy is not routinely recommended for unifocal or multifocal low-risk PTMC patients, as it has not been shown to impact recurrence rates (7, 21, 22). Recently, the ESTIMABL2 trial, the first large prospective randomized study involving low-risk patients with differentiated thyroid cancer (mean largest tumor diameter of 13.4–13.7 mm), demonstrated the non-inferiority of omitting RAI ablation in terms of functional, structural and biological outcomes at 3 years (23).

The aim of the present study was to investigate the approach of Greek endocrinologists, members of the Hellenic Endocrine Society (HES), toward AS and the management of low-risk PTMC and to compare these practices with those recommended in the current clinical practice guidelines by conducting web-based survey using clinical scenarios.

## Materials and methods

### Study design and recruitment

The study targeted the members of HES. At the time of the end of the survey, HES numbered 809 members with an equal sex distribution (433 women). The target sample size was 162 participants, comprising 20% of the study population. A web-based survey constructed using Survey Legend, a survey web application which ensured anonymous collection of the responses and automatic exclusion of repeated submissions from the same IP address, was employed. The HES emailed to all its members the link to the online questionnaire platform along with a brief description of the study objectives. Participants were assured that participation was voluntary, with confidentiality and anonymity strictly maintained. The contact details of the principal investigator were provided for clarification purposes.

Recruitment occurred between November 2023 and April 2024, with the first email sent in November 2023 and a reminder email sent on January 24, 2024. Before the main survey, a pilot study was conducted among a small group of endocrinologists (*n* = 15). These participants completed the questionnaire twice, with a 5-month interval between responses, to assess its repeatability using Cohen’s Kappa test. Data collected during this pilot phase were excluded from the final analysis.

The questionnaire was designed to explore the clinical approach of Greek endocrinologists to the management of thyroid nodule and low-risk papillary thyroid carcinomas. It consisted of three parts. The first part included seven questions about demographic data of the respondents (sex, age, occupational status, geographical region and years of experience), self-reported confidence in diagnosis and treatment of patients with thyroid nodule or cancer (on a 5-point Likert scale) and their choice of the most helpful educational tools for managing thyroid nodules and thyroid cancer (conferences, clinical practice or literature). The second part presented 12 clinical scenarios regarding the management of thyroid nodules with indeterminate cytology and low-risk papillary thyroid carcinomas requiring endocrinologists to choose among four possible management approaches. The third part included a final question about the primary reasons for nonadherence to guidelines, allowing respondents to select more than one answer. In the present study, the answers of two clinical scenarios involving a 60-year-old woman with papillary thyroid very-low-risk microcarcinoma are analyzed and presented (Box 1 and Supplementary data (see section on Supplementary materials presented at the end of the article)).

Box 1 **The two clinical scenarios submitted to participants.**

1. A 60-year-old woman has 7 mm papillary microcarcinoma, intraparenchymal and without suspicious cervical lymph nodes on ultrasound (low-risk). The patient has no comorbidities. What would you recommend?

A. Active surveillance

B. Thermal ablation (minimally invasive treatment)

C. Lobectomy

D. Total thyroidectomy

2. A 60-year-old woman has undergone total thyroidectomy for a classical papillary 7 mm microcarcinoma and has no known infiltrated cervical lymph nodes, vascular infiltration or extrathyroidal extension (low-risk). How likely are you to recommend radioactive iodine ablation?

A. Very likely

B. Likely

C. Less likely

D. Unlikely

### Ethics approval and consent to participate

Ethical approval was gained by the Ethics Committee of the University of Patras (approval number 15770/28-07-2023). Moreover, standard ethical requirements were rigorously applied: informed consent was received by all participants, anonymity and the right to withdraw from the study at any point was retained. All methods were carried out in accordance with the ethical guidelines of the Declaration of Helsinki as revised in 2013.

### Data analysis

Descriptive statistics were computed to summarize demographic data. Group proportions were calculated for categorical variables and their differences were tested using χ^2^ or Fisher’s exact tests. Spearman rho correlation coefficient was calculated to assess bivariate associations. Group comparisons of numerical variables were performed by the nonparametric Mann–Whitney U test. Multivariate binary (each response choice versus all other available choices) logistic regression models were constructed with the forward stepwise (likelihood ratio) method to estimate associations with participant characteristics (sex, years since residency, average number of patients with differentiated thyroid cancer managed annually, clinician practice location, employment sector and self-confidence level). The statistical significance level was set at *α* = 0.05, and analyses were conducted in the IBM SPSS v.28.

## Results

Twenty-five percent (201/809) of the total members of the HES filled in and submitted the questionnaire.

### First part: demographic characteristics

The demographic characteristics of the participants are presented in Table 1. Age, sex, working in the private or public sector and the geographical distribution of the respondents were representable of the distribution of the total members of the HES (Kolmogorov–Smirnov Z test, *P* = 1.000). The inter-rater reliability of the questionnaire was substantial to excellent (kappa = 0.804, *P* < 0.001).

**Table 1:** Demographic characteristics of the study population (endocrinologists – members of the Hellenic Endocrine Society). Data are presented as percent or as the mean ± SD.

Characteristics	Values
Total *n*	201
Sex	
Male	42.0
Female	58.0
Age range (years)	
30–39	10.4
40–49	31.3
50–59	35.8
60–69	18.9
>70	3.5
Employed in	
Public sector	23.4
Private sector	76.6
Years since residency	
1–5	17.9
6–10	16.4
11–30	56.7
>30	9.0
Location of clinical practice	
Two largest cities (Athens, Thessaloniki)	68.2
Other areas	31.8
Level of self-confidence*	
Moderately confident	27.0
Very confident	61.5
Absolutely confident	11.5
Patients with DTC managed annually, *n*	11.7 ± 17.8

DTC, differentiated thyroid cancer.

* Level of self-confidence in managing patients with thyroid nodules or cancer.

### Second part: responses to the two clinical scenarios

**Figure 1:**
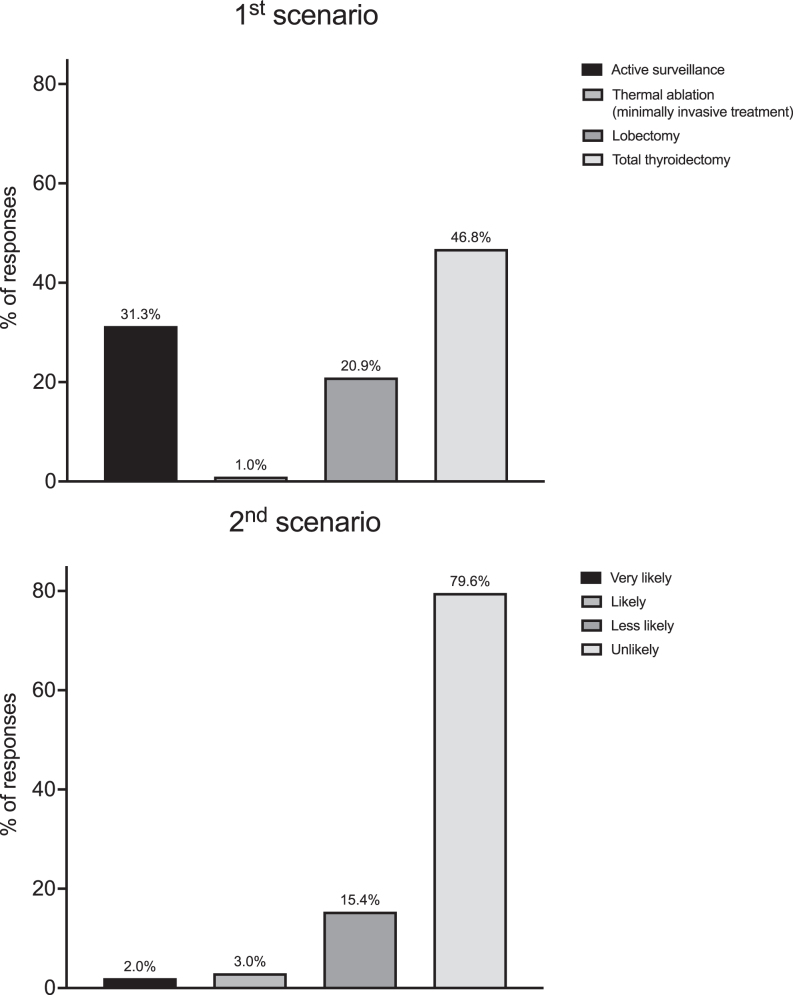
Distribution of physicians’ responses to two clinical scenarios. When queried about the management of low-risk PTMC (first scenario), almost half of the surveyed HES members (46.8%) answered total thyroidectomy as the most appropriate management option, followed by active surveillance (31.3%), lobectomy (20.9%) and thermal ablation (1%). In the second scenario, the vast majority (95%) considered unlikely/less likely with the remaining responders (5%) answering that it is likely/very likely to proceed with RAI therapy after total thyroidectomy a low-risk PTMC.

In the first scenario, almost half of the surveyed HES members (*n* = 94, 46.8%) chose total thyroidectomy as the most appropriate management option for low-risk PTMC in a 60-year-old woman, followed by AS (*n* = 63, 31.3%) and lobectomy (*n* = 42, 20.9%), and 1.0% selected thermal ablation. In the second scenario, the vast majority (*n* = 160, 79.6%) were unlikely to use RAI ablation and another 15.4% (*n* = 31) less likely to do so. The remaining responders reported that they were likely/very likely to proceed with RAI ablation (*n* = 10, 5.0%) (Fig. 1).

In both clinical scenarios, multivariate binary logistic regression models (each option versus all others) revealed no significant associations of age, sex, self-confidence level, private or public sector employment, years of experience, average annual number of patients with differentiated thyroid cancer and location of clinical practice on decision-making regarding patient management. Thus, the preference for AS over any other intervention was not associated with any personal or occupational trait of the participants. Furthermore, when we separately examined endocrinologists who chose lobectomy versus those who chose total thyroidectomy in the first scenario, no association of their choice with any demographic, occupational or personal characteristic of the endocrinologists was observed.

### Third part: reasons for noncompliance with international guidelines

In this final question, Greek endocrinologists were asked to provide one or more reasons for noncompliance with international guidelines regarding the management of both low-risk papillary thyroid carcinomas and thyroid nodules. Among endocrinologists, 46.8% expressed skepticism toward the recommendations of scientific societies but also cited other reasons, such as the inability to perform reliable ultrasound examinations (35.3%), limited access to molecular tests (34.3%), insufficient information (22.4%) and a shortage of experienced surgeons (17.9%).

## Discussion

This is the first known survey conducted in Europe, examining the attitudes of Greek endocrinologists toward AS and the management of low-risk PTMCs. The high survey response rate and the comparable demographic data between survey participants and the overall membership of the HES suggest that the surveyed sample is highly representative of the HES members. The main finding of our survey is that the two-thirds of the Greek surveyed endocrinologists favored immediate surgery, mainly total thyroidectomy, over AS, while the remaining one-third preferred AS.

These results align with a Brazilian survey, where fewer than 25% of respondents recommended AS in PTMC cases without additional risk factors (24). The majority favored immediate surgery, mostly total thyroidectomy. A similar survey in Australia and New Zealand reported that one-third of surveyed endocrinologists supported AS as an alternative to immediate surgery (25). However, the small sample size (27 endocrinologists) limits the generalizability of these findings. Notably, in the same study, surgeons were more likely to adopt AS compared to endocrinologists (52% vs 37%), particularly those who could themselves perform thyroid ultrasounds. A survey of Japanese endocrine surgical centers reported that nearly all respondents (96%) indicated that they would discuss AS with their patients (26). However, only 31% of respondents would actively recommend AS, while 26% would prefer surgery and 39% would leave the decision to the patient. In a recent U.S. survey, the 76% of endocrinologists, general surgeons and otolaryngologists responded that AS is an appropriate option for select thyroid cancer patients (27). However, when questioned about whether they currently recommend AS in their own practice, 44% responded ‘yes’ and 56% responded ‘no’. Notably, among those who answered yes, 29% would not even suggest AS for a case scenario like ours, i.e. a 60-year-old female with a <1 cm tumor and no lymph node involvement. These data underscore the substantial barriers to the broader adoption of AS as a management strategy for low-risk thyroid cancers.

The Australian and the U.S. surveys examined the barriers to implementing AS (25, 27). The most common barrier reported was patients’ reluctance to choose AS over surgery, influenced by the emotional impact of a cancer diagnosis. Previous research supports this notion, showing that patients often opt for surgery despite potential complications (28). Other barriers included lack of patient compliance, limited access to follow-up care, misclassifying risk levels, uncertainty about the duration of follow-up and a lack of clear strategies for managing AS (25, 27). In our study, we investigated the reasons for noncompliance with high-quality international guidelines released over the past decade among endocrinologists, rather than exploring patient preferences. It is important to note that the final question in our survey addressed the management of both thyroid nodules and low-risk papillary thyroid carcinomas, which constitutes a limitation of our study. Nearly half of the respondents expressed skepticism about implementing the recommendations of scientific societies, likely due to a shortage of resources and a lack of familiarity with the guidelines. To enhance the adoption of AS approach, both physicians and patients require further education and improvements to the sociomedical environment. In addition to the above barriers, one can also take into consideration the deeply ingrained habitual clinical practice behaviors, which present a significant barrier to adopting new, evidence-based therapeutic approaches (29). This is evident not only from the preference of the surveyed Greek endocrinologists for immediate surgery over AS, but also from their preference for choosing total thyroidectomy over lobectomy, which remains the standard practice in Greece even for low-risk PTMC, despite guidelines for over a decade suggest lobectomy to be sufficient for such patients (7).

Thermal ablation has emerged as a viable treatment option for low-risk PTMC, providing an alternative to AS and immediate surgery (30). Thermal ablation showed no tumor progression over a pooled 5-year follow-up, and no patients required delayed surgery due to anxiety in a recent review, which contrasts with the 8–32% of patients undergoing surgery after AS due to similar concerns (31). However, thermal ablation’s usage remains limited for several reasons, including its novelty, the ongoing evaluation of long-term outcomes and concerns over safety and efficacy. Furthermore, there is currently limited number of experienced specialists in thermal ablation techniques in Greece, influencing the acceptance of thermal ablation from the surveyed practitioners.

The choice of total thyroidectomy over lobectomy for managing PTMC can be determined by several factors, despite the growing trend toward more conservative surgical options (32). One reason is the perception that total thyroidectomy offers a more definitive treatment and reduces the need for further surgeries if contralateral nodules or lymphadenopathy or aggressive features are discovered after lobectomy. Furthermore, individual surgeon preferences, interpretations of clinical guidelines and personal experience can lead to variations in surgical approaches (32). In our view, the most important factor is that the historical precedent, where total thyroidectomy has long been considered the standard approach for thyroid cancer, despite the convincing literature supporting the safety and efficacy of lobectomy. Although the data supporting these considerations comes from surgical studies, we believe that same factors influence endocrinologists’ attitudes toward total thyroidectomy for PTC (33).

There is a growing consensus and evidence suggesting that RAI remnant ablation is not recommended for low-risk DTC patients (7, 22) and the alignment of endocrinologists' practices with these recommendations has shown notable changes over the past decade (34, 35). Almost all of the surveyed Greek endocrinologists responded that it is (very) likely not to use RAI remnant ablation in the second scenario, in line with current recommendations. Although, significant variations in practice remain, indicating that not all endocrinologists fully align with the recommendations in every thyroid cancer case, this does not apply in the case of unifocal T1a tumors in the absence of any additional risk factors, where full consensus for omitting RAI remnant ablation was found (36).

While our study yields significant findings regarding the attitudes of Greek endocrinologists toward AS for low-risk PTMC, we acknowledge certain limitations that warrant further exploration. This study also forms part of a broader survey examining the management of thyroid nodules with Atypia of Undetermined Significance (AUS) cytology and low-risk papillary thyroid carcinomas bigger than 1 cm. Consequently, the last question in the third part is general in nature and captured general attitudes and preferences, which, while informative, do not investigate in depth the underlying reasons behind the results. Besides skepticism about the evidence expressed by half of the participants, about one-third reported an inability to perform reliable ultrasound, about one-third cited the unavailability of molecular tests and about one-fifth indicated a lack of proper information or a shortage of high-volume surgeons. Future studies should incorporate more detailed questions to assess these aspects, including the experience of using diagnostic tools such as ultrasound, the impact of practice settings and the level of familiarity with AS guidelines. Such investigations would help identify specific strategies to promote the adoption of AS in clinical practice.

In conclusion, AS for managing very low-risk PTMCs is currently adopted by only one-third of Greek endocrinologists. This aligns with the practices of other national societies but shows a notable discrepancy with high-quality international clinical guidelines for managing very low-risk PTMCs. Targeted efforts are needed to bridge this gap, including improved access to diagnostic tools, enhanced training, educational initiatives to build trust in evidence-based approaches and adequate time for these changes to take effect. We hope that insights from our web-survey will contribute to reducing overtreatment and adoption of the upcoming ATA guidelines, alleviating unnecessary patient burdens and optimizing outcomes in the management of low-risk PTMCs.

## Supplementary materials



## Declaration of interest

The authors declare that there is no conflict of interest that could be perceived as prejudicing the impartiality of the work reported.

## Funding

This research did not receive any specific grant from any funding agency in the public, commercial or not-for-profit sector.

## Author contribution statement

GE: conceptualization (supporting); ES: methodology (lead), formal analysis (lead); OK: conceptualization (supporting); KS: conceptualization (lead); MM: conceptualization (lead), project administration, supervision (lead). All authors have been involved in drafting the manuscript and revising it critically for important intellectual content. All authors read and approved of the final manuscript.

## Ethics approval

Ethics approval was granted by the Ethics Committee of the University of Patras (approval number 15770/28-07-2023).
