# Supplementary material for: Active surveillance for low-risk papillary thyroid microcarcinoma: a web-survey on clinician readiness for change
Source: Eur Thyroid J. 2025 Mar 17;14(2):e250013. doi: 10.1530/ETJ-25-0013 (PMC11949526; doi:10.1530/ETJ-25-0013)
Supplement: Supplementary file 1 [file supplementary_materials.pdf]

1st part: Demographic data

1. Sex

a. Male

b. Female

c. Other

2. Age (years)

a. 30-39

b. 40-49

c. 50-59

d. 60-69

e. >70

3. Years since obtaining an endocrinology specialty

a. 1-5

b. 6-10

c. 11-30

d. >30

4. What is your workplace?

a. Private sector

b. Public sector

5. In which Prefecture do you practice your specialty? …………………………………………….

6. How confident do you feel about the management of patients with thyroid nodule or papillary thyroid carcinoma?

a. Not at all confident

b. A little bit confident

c. Moderately confident

d. Very confident

e. Absolutely confident

7. Which of the following do you think would help you manage patients with thyroid nodule or papillary thyroid carcinoma?

a. Conferences

b. Clinical tutorials - case studies in tertiary hospitals

c. Bibliography

2nd part: Clinical scenarios

1. A 60-year-old woman has 7 mm micropapillary carcinoma, intraparenchymal and without suspicious cervical lymph nodes on cervical ultrasound (low risk). The patient has no co-morbidities. What would you recommend?

A. Active surveillance

B. Thermal ablation (minimally invasive treatment)

C. Lobectomy

D. Total thyroidectomy

2. In a 60-year-old woman who has undergone total thyroidectomy for classical papillary 7mm carcinoma and has no known infiltrated cervical lymph nodes, vascular infiltration or extrathyroid expansion (low-risk). Would you give Radioactive Iodine ablation?

A. Very likely

B. likely

C. Less likely

D. Unlikely

3rd part: Exploring Reasons for non-adherence

What is/are the main reason(s) for non-adherence to the guidelines regarding thyroid nodules or low-risk papillary thyroid carcinomas?

A. Insufficient information

b. I am not convinced by the guidelines, and I am concerned about my patient's safety

c. Inability to perform a reliable neck ultrasound

D. Inability to conduct molecular testing

e. lack of experienced surgeons across Greece
